# Overexpression of synapsin Ia in the rat calyx of Held accelerates short-term plasticity and decreases synaptic vesicle volume and active zone area

**DOI:** 10.3389/fncel.2013.00270

**Published:** 2013-12-20

**Authors:** Mariya Vasileva, Robert Renden, Heinz Horstmann, Daniel Gitler, Thomas Kuner

**Affiliations:** ^1^Institute of Anatomy and Cell Biology, Heidelberg UniversityHeidelberg, Germany; ^2^Department of Physiology and Cell Biology, Faculty of Health Sciences and Zlotowski Center for Neuroscience, Ben-Gurion University of the NegevBeer-Sheva, Israel

**Keywords:** synapse, synaptic transmission, vesicle cluster, active zone, short-term depression

## Abstract

Synapsins are synaptic vesicle (SV) proteins organizing a component of the reserve pool of vesicles at most central nervous system synapses. Alternative splicing of the three mammalian genes results in multiple isoforms that may differentially contribute to the organization and maintenance of the SV pools. To address this, we first characterized the expression pattern of synapsin isoforms in the rat calyx of Held. At postnatal day 16, synapsins Ia, Ib, IIb and IIIa were present, while IIa—known to sustain repetitive transmission in glutamatergic terminals—was not detectable. To test if the synapsin I isoforms could mediate IIa-like effect, and if this depends on the presence of the E-domain, we overexpressed either synapsin Ia or synapsin Ib in the rat calyx of Held via recombinant adeno-associated virus-mediated gene transfer. Although the size and overall structure of the perturbed calyces remained unchanged, short-term depression and recovery from depression were accelerated upon overexpression of synapsin I isoforms. Using electron microscopic three-dimensional reconstructions we found a redistribution of SV clusters proximal to the active zones (AZ) alongside with a decrease of both AZ area and SV volume. The number of SVs at individual AZs was strongly reduced. Hence, our data indicate that the amount of synapsin Ia expressed in the calyx regulates the rate and extent of short-term synaptic plasticity by affecting vesicle recruitment to the AZ. Finally, our study reveals a novel contribution of synapsin Ia to define the surface area of AZs.

## Introduction

Synapsins are a family of abundant neuron-specific phosphoproteins whose expression varies across synapse types and brain regions with synapsin Ia, Ib, IIa, IIb, and IIIa being most abundant (De Camilli et al., 1983; Sudhof et al., 1989; Matus-Leibovitch et al., 1995, 1997; Hosaka and Sudhof, 1998). They associate with the cytoplasmic surface of SVs in a phosphorylation-dependent manner and dimerize to immobilize SVs within synapses (Greengard et al., 1993; Hilfiker et al., 1999; Hosaka and Sudhof, 1999). Synapsins are thought to tightly control the reserve pool of vesicles, which is required to sustain neurotransmitter release in response to repetitive neuronal activity (Llinas et al., 1985; Pieribone et al., 1995; Rosahl et al., 1995; Ryan et al., 1996). Perturbing synapsin function leads to decreased SV numbers within nerve terminals, decreased recycling pool of SVs, and altered short-term synaptic plasticity (Rosahl et al., 1993; Pieribone et al., 1995; Takei et al., 1995; Hilfiker et al., 1998; Humeau et al., 2001; Feng et al., 2002; Gitler et al., 2008; Vasileva et al., 2012).

Formation of homo- and heterodimers depends on isoform composition and is required for synapsin localization to the presynaptic terminals. Different synapsin isoforms have different propensities to dimerize, affecting SV targeting to presynaptic terminals (Gitler et al., 2004a). The synapsin Ia isoform forms homodimers and is robustly targeted to SVs, thereby cross-linking them (Hosaka and Sudhof, 1999). The splice variant synapsin Ib lacks the SV-targeting E-domain and therefore depends on heterodimer-formation with other isoforms for proper targeting to the presynaptic terminal (Gitler et al., 2004a). Synapsin IIa, which is properly targeted to SVs, is the only isoform that can partially rescue a severe short-term depression phenotype in synapsin-null hippocampal neurons (Gitler et al., 2008). Therefore, it is important to understand the role of synapsin isoforms in the organization of reserve pool vesicles *in vivo* at central nervous system synapses.

We characterized the contribution of synapsin I isoforms to SV distribution and synaptic transmission during and following high-frequency activity. For this purpose we overexpressed synapsin I isoforms fused to fluorescent reporters, to manipulate SV clustering and/or mobility within the calyx of Held, a giant terminal in the auditory brainstem circuit mediating binaural sound localization (Borst and Soria van Heave, 2012). This synapse provides a well-suited model system to investigate SV protein organization and function in close proximity to the release sites. The calyx of Held harbors more than 600 active zones (AZ) (Sätzler et al., 2002; Dondzillo et al., 2010) and ~3,000–4,000 vesicles released upon direct strong Ca^2+^ stimuli (Neher and Sakaba, 2001; Sakaba and Neher, 2001a; Sun and Wu, 2001); for a review see (Rizzoli and Betz, 2004; Schneggenburger and Forsythe, 2006). At postnatal day (P) 16, after the onset of hearing, the calyx has nearly reached an adult stage, and successfully mediates high-frequency synaptic transmission (Renden et al., 2005; Sonntag et al., 2011; Borst and Soria van Hoeve, 2012). Synapsins Ia, Ib, IIb and IIIa are present in the calyx of Held at his maturation stage. Surprisingly, the IIa isoform—shown to sustain repetitive transmission in glutamatergic terminals (Gitler et al., 2008)—could not be detected in the calyx.

Overexpression of both synapsin I isoforms at the calyx of Held resulted in a redistribution of SVs within the presynaptic terminal, leading to an increased short-term depression in response to high frequency stimulation trains and faster recovery. Electron microscopy analysis showed overexpression of synapsin Ia led to decreased SV volume and AZ area. The number of SVs clustered in close proximity to AZs was reduced while the total SV number within the presynaptic terminal remained unchanged. Hence, we conclude that synapsin I isoforms—and synapsin Ia in particular—affect short-term plasticity by facilitating activity-dependent release and acceleration of SV refilling following high-frequency activity (de Lange et al., 2003; Rizzoli and Betz, 2004; Denker and Rizzoli, 2010).

## Materials and methods

### Plasmid cloning and virus preparation

Chimeric recombinant adeno-associated viruses combining capsids of serotype 1 and serotype 2 (rAAV1/2) were used for expressing synapsin isoforms in the medial nucleus of the trapezoid body (MNTB). Plasmids expressing synapsin isoforms, tagged at the N-terminus with enhanced green/yellow fluorescent proteins (EGFP/EYFP), were constructed as follows: EGFP, EYFP-synapsin Ia, or synapsin Ib was excised from pEGFP, EYFP-synapsin Ia, or synapsin Ib-C1, respectively, (Chi et al., 2001, 2003; Gitler et al., 2004a; Bonanomi et al., 2005; Valente et al., 2012) via NheI/SpeI restriction endonucleases and inserted into pAM-AAV (MfeI/SpeI) containing the 1.1 kb cytomegalovirus enhancer element, chicken ß-actin promoter, the woodchuck post-transcriptional regulatory element (WPRE) and the bovine growth hormone polyA (bGH), to generate pAM-CBA-EYFP, EGFP-synapsin Ia, and synapsin Ib-WPRE-bGH. Membrane-targeted green fluorescent protein (mGFP) has been described previously (Dondzillo et al., 2010). Recombinant AAV chimeric virions containing a 1:1 ratio of AAV1 and AAV2 capsid proteins and the foreign gene were generated as described (Grimm et al., 2003; Klugmann et al., 2005). Viral solution was purified on heparin columns, kept at 4°C and used within 4 weeks.

### Animals

Experiments were conducted in accordance with the German animal welfare guidelines (TierSchG) and were approved by our local animal care and use committee (Regierungspräsidium Karlsruhe). Sprague-Dawley rats from either sex were used for all experiments. Seven rats were injected with mGFP for detection of endogenous synapsin isoforms. Four rats were co-injected with mGFP and EYFP-synapsin Ia, five rats co-injected with mGFP and EYFP-synapsin Ib, and five rats injected with mGFP were used for fluorescent structural analysis. Seven rats injected with EYFP-synapsin Ia, 14 rats injected with EYFP-synapsin Ib and 22 non-injected wild type rats (controls) were used for electrophysiology. Two rats injected with EGFP-synapsin Ia and two rats injected with EGFP were used for electron microscopy (EM) analysis. With regard to immunohistochemical and morphological analyses, we define rats expressing mGFP/EGFP as control rats (CTRL) to which we compare rats overexpressing EYFP-synapsin Ia, or synapsin Ib. P16–18 animals were used to circumvent the strong myelination present in the brain stem of adult rats, which makes *in vitro* electrophysiological recordings impossible in the adult MNTB. Although at P16 the auditory system still undergoes maturational changes, the calyx of Held has achieved robust functional properties (Borst and Soria van Heave, 2012).

### Stereotaxic injection

rAAV1/2 particles were delivered to the anterior ventral cochlear nucleus (aVCN) of rats at postnatal day 6 via stereotaxic injection as reported previously (Wimmer et al., 2004), but using isofluorane inhalation anesthesia (Dondzillo et al., 2010; Schwenger and Kuner, 2010). For aVCN injection, ~ 2 μl viral solution was distributed among 6 injection sites with the following Cartesian coordinates relative to the bregma and the midline, respectively, (in mm): −8.8; 1.15, −8.5, 1.15; −8.2, 1.10; −8.7, 1.00; −8.4, 0.95; −8.1, 0.95. At each one of these *x*, *y* positions injections were made at depth (*z*) of 0.45 mm ventral from the bregma and axially (A_*x*_)at −8.0 to −9.5 mm.

### Immunohistochemistry

Ten days after injection, rats were anesthetized with a lethal dose of sodium-pentobarbital (narcoren; 70.4 mg/kg body weight) and transcardially perfused with 40 ml phosphate-buffered saline (PBS, in mM 136.89 NaCl, 2.68 KCl, 9.59 Na_2_HPO_4_, 1.47 KH_2_PO_4_), followed by 40 ml 4% paraformaldehyde in PBS. The brains were extracted and post-fixed in 4% paraformaldehyde overnight at 4°C. The rat brain stem was embedded in 3% agarose and 50 μm-thick coronal sections were cut on a vibratome (HR-2, Sigmann Elektronik). Prior to staining, sections were washed 3 × 10 min in PBS and incubated 60 min at room temperature (RT, 23–25°C) in PBS containing 5% normal goat serum and 1% TritonX-100. The primary antibodies were diluted in 4°C PBS with 1% normal goat serum and 0.5% TritonX-100, and incubated overnight at RT. The secondary antibodies, conjugated to Alexa fluorophores (Molecular Probes; Darmstadt, Germany), were diluted in the same buffer as primaries, and incubated for 90 min at RT in the dark. After 3 × 10 min washing in 4°C PBS, the sections were rinsed with distilled H_2_O and embedded in SlowFade^®^ Gold antifade reagent (Invitrogen; Darmstadt, Germany). In case of synapsin IIIa, normal goat serum was exchanged with fetal bovine serum (Pan Biotech GmbH; Aidenbach, Germany).

### Antibody characterization

None of the synapsin antibodies gave rise to detectable signals in synapsin triple knock-out mice (Gitler et al., 2004b), and data not shown). Additionally, synapsin IIb antibody detected EGFP-synapsin IIb expressed in HEK cells. All antibodies penetrated throughout the entire 50 μm-thick slices. Synapsin I antibody (1:1000 dilution) was purchased from Synaptic Systems (Göttingen, Germany). Synapsin II antibody (1:1000 dilution) was obtained from Abcam (Cambridge, UK). Synapsin IIIa antibody (1:50 dilution) was purchased from Santa Cruz Biotechnology (Heidelberg, Germany). vGluT1 antibody (1:1000 dilution) was obtained from Millipore (Billerica, MA). Polyclonal rabbit antibodies raised against synapsin Ib (1:1000 dilution, clone G-278, peptide from the C-terminus of synapsin Ib: K-A-S-P-A-Q-A-Q-P), synapsin IIa (1:500 dilution, clone G-281, a.a. 470–484 from domain H from synapsin IIa), synapsin IIb (1:1000 dilution, clone G-466/467, peptide from C-terminal I-domain of synapsin IIb) and synapsin E-domain (1:2500 dilution, clone G-304, a.a. 680–704 from C-terminal E-domain of synapsin Ia) were kindly provided by P. Greengard's lab.

### Confocal imaging and dye separation

Images were acquired on a Leica TCS SP5 microscope equipped with a 63x glycerol-immersion objective (N.A. 1.3) and 476 nm, 488 nm, 514 nm, 568 nm and 647 nm laser lines to excite the respective fluorophores. After collecting images with a voxel size of 91.71 nm × 91.71 nm axial and 150 nm vertical dimensions, data was deconvolved using Huygens software (Scientific Volume Imaging). In experiments involving imaging of mGFP and EYFP in the same slice preparation, spectral unmixing of the acquired data was performed via the Channel Dye Separation Application of the Leica Application Software, prior to deconvolution. For the purpose of defining reference regions we imaged terminals expressing either mGFP or EYFP-synapsin Ia/Ib. Calyces in the ipsilateral (non-infected) MNTB were used as a reference for vGluT1 staining. Reference and experimental images were acquired with the same microscope settings.

### Excision of calyx-specific immunosignals and visualization of 3D image data

Excision of calyx-specific immunosignals and 3D reconstruction of image data was performed with the visualization software AMIRA 4.1.1 (Visage Imaging) as described previously (Dondzillo et al., 2010). Briefly, the mGFP channel was segmented applying a threshold to select only voxels belonging to the calyx, generating a 3D image mask. Next, the mask was applied to the channel containing the immunosignal or EYFP-synapsin Ia/Ib, generating a 3D image delineated by the calyx volume. Volumes of structures within the calyx were then calculated and compared between different treatments.

### Electrophysiology

Acute transverse 200 μm-thick slices were made from P16–18 rat brainstem, containing intact ventral stria and MNTB. Slices were made on a vibratome (HM650V, Microm, Walldorf, Germany), at 4°C. Slicing media contained (in mM): 85 NaCl, 2.5 KCl, 25 glucose, 25 NaHCO_3_, 1.25 NaH_2_PO_4_, 75 sucrose, 3 3-myo-inositol, 2 Na pyruvate, 0.4 Ascorbic Acid, 0.5 CaCl_2_, and 7 MgCl_2_. After bubbling with carbogen (95% O_2_, 5% CO_2_), the pH was 7.3 and density was 315–320 mOsm. Slices were incubated in normal artificial cerebrospinal fluid (aCSF) for ~ 60 min at 35°C, then used thereafter at RT. Normal aCSF contained (in mM) 125 NaCl, 2.5 KCl, 25 glucose, 25 NaHCO_3_, 1.25 NaH_2_PO_4_, 2 CaCl_2_, and 1 MgCl_2_, pH 7.3 after bubbling with carbogen, density 315–320 mOsm. Strychnine (0.5 μM), SR9551 (10 μM), and APV (50 μM) were also added to the recording bath solution, which perfused the slice at 1-2 mL/min. All materials were purchased from Sigma Aldrich, unless stated otherwise.

MNTB cells were visualized at 60x, using an EM CCD camera (Sensicam EM, PCO, Kelheim Germany), and gradient contrast optics on a Zeiss microscope (Axioskop 2 plus, Carl Zeiss, Oberkochen Germany). Infected calyx terminals were identified by strong fluorescence signal opposing intact principal cell somata. MNTB cells were additionally preselected based on the presence of extracellular presynaptic and postsynaptic waveforms during afferent stimulation, showing intact axonal connectivity (Borst et al., 1995; Wu and Kelly, 1993).

Whole-cell patch clamp recordings from the MNTB principal cell were made using 1.5 mm outer diameter filamented borosilicate glass (WPI, Sarasota FL), pulled to tip resistances of 1.8–2.5 MΩ on a Sutter P-97 puller (Sutter, Novato CA). Pipette internal solution consisted of (in mM): 130 Cs-gluconate, 10 CsCl, 5 Na_2_ phosphocreatine, 10 HEPES, 5 EGTA, 10 TEA-Cl, 4 Mg-ATP, 0.3 GTP, pH 7.2, 305-310 mOsm. Cells were clamped at −70 mV, unless excitatory postsynaptic current (EPSC) amplitude exceeded 18 nA, in which case holding potential was reduced to -30 mV to reduce the driving force. Because the recordings of evoked EPSCs (eEPSC) were made at multiple holding potentials, direct comparison is unwarranted without biased selection. To assist in cross-cell normalization, EPSCs were evoked in multiple cells at both *V*_hold_ −70 and −30 mV holding potential (data not shown). From the resulting IV relationship a scaling factor of 0.025 ± 0.001 nA /mV for the eEPSCs amplitude was calculated, and applied to generate a predicted eEPSC magnitude at *V*_*hold*_ -70 mV. Access resistance was in all cases ≤6 MΩ, and compensated 85–94%, such that residual series resistance (Rs) <0.5 MΩ. Leak current was usually less than 100 pA, and in no cases exceeded 200 pA.

EPSCs were evoked via midline stimulation using parallel Pt-Ir bipolar electrodes (200 μm distance; FHC, Bowdoin ME). Stimuli were applied with an optically isolated bipolar stimulator (NPI Electronic GmbH, Tamm Germany), using 100 μs duration pulses. Stimulation amplitude was set to 1 V above threshold (1.0–3.5 V). Stimulus trains were generally repeated 3–5 times per cell, with a 30 s interval between trains, and the mean initial EPSC magnitude was normalized. The paired-pulse ratio (PPR), rate of depression, and steady-state level of the depressed synapse were then measured per cell, from the averaged traces (von Gersdorff and Borst, 2002). Receptor desensitization is not present at stimulation frequencies <300 Hz at the calyx of Held synapse at this age, so blockers of desensitization (e.g., cyclothiazide) were not needed (Renden et al., 2005).

Quantal content of evoked events was determined as the quotient of the charge integral of a single eEPSC over the median quantal charge, measured per cell. The size of the readily-releasable pool (RRP) was estimated according to (Schneggenburger et al., 1999; Stevens and Williams, 2007), using a linear regression of the cumulative EPSC peaks from the 30th–50 pulses in a train at 300 Hz, where the RRP should be fully depleted (data not shown). The Y-intercept of this fit yields the size of the releasable pool, whereas the slope is proportional to the steady state. The number of SVs in the RRP was calculated as the quotient of the intercept value and the median quantal amplitude from recordings in the same cell.

Data were acquired with a HEKA EPC10 amplifier, driven by a PC running Pulse 8.80 software (HEKA, Lambrecht/Pfalz Germany). Data were filtered on-line at 2.9 kHz. Data were corrected off-line for voltage errors due to uncompensated series resistance (Traynelis, 1998). Data analysis was performed in Igor 6.0 software (Wavemetrics, Lake Oswego Oregon USA). Detection of spontaneous quantal events (spEPSC) was performed using a threshold-based detection algorithm in Igor (Wimmer et al., 2004), with detection threshold set to 14 pA. Detected events were excluded if they had decay times <0 or >5 ms, rise times <0, charge <0. Events larger than 500 pA were also excluded from analysis, as these likely represented multiquantal events.

### Photooxidation and electron microscopy

Photooxidation of EGFP and EGFP-fusion proteins was performed according to (Horstmann et al., 2013). Briefly, EGFP or EGFP-synapsin Ia virus-injected animals were perfused as described above and 100 μm-thick sections containing infected MNTB regions were washed for 10 min consecutively in PBS and in 50 mM Tris-HCl, pH 7.6. Sections were bubbled for 60 min with pure O_2_ in 50 mM Tris-HCl at 4°C and stored overnight in a closed system. On the following day, the sections were again exposed to pure O_2_ in 50 mM Tris-HCl for 60 min at 4°C, followed by 10 min incubation in 5 ml ice-cold 50 mM Tris-HCl solution containing 5 mg diaminobenzidene (DAB) and 40 μl 1 M NaOH (pH 7.6). A Leica DM IRB microscope equipped with a 40x oil immersion objective (NA: 0.75–1.25) was used for photooxidation. The light path aperture was closed to minimum and had a diameter of 150 μm. The region of interest was illuminated with wavelength 488 nm for 5 min with NA 0.75, followed by 5 min with NA 1.0 and then by 5 min with NA 1.25 and fine precipitates were observed in the exposed area. The tissue was then washed 2× in 50 mM Tris-HCl and incubated in 0.1 M cacodylic acid buffer buffer pH 7.4. The MNTB area containing the photooxidized terminals was dissected out and post-fixed in a mixture of 2% potassium potassium ferrocyanide and 2% osmium tetroxide for 120 min. After 3 washing steps in distilled water they were dehydrated in an ascending alcohol series and stored overnight in a 1:1 mixture of propylene oxide and epoxy (Serva; Heidelberg, Germany). The tissue was then transferred into fresh resin and polymerized at 60°C for 36 h. After polymerization we cut 20–30 serial sections per sample with a thickness of ~ 35 nm on an ultramicrotome. The resulting ribbons were attached to a silicon wafer (Si-Mat; Silicon Materials, Germany), counterstained with uranyl acetate and lead citrate, and prepared for imaging following published procedures (Horstmann et al., 2012). SEM imaging was done on a LEO Gemini 1530 with an inlens detector with a working distance of 1.8 mm, a 60 μm aperture and 3 KeV acceleration voltage. We used 10,000× magnification. An 11.2 × 8.4 μm area of the specimen was digitized at 3072 × 2304 pixels with pixel dwell time of 40 μs. Pre-alignment was done manually on the s.e.m. by correlating the previous image with the next via stage rotation and shifting the electron beam. Section thickness was measured *post-hoc* according to (Reid and Beesley, 1991; Horstmann et al., 2012). Briefly, sections were mounted on grids in the usual way and then the complete grid was re-embedded. After polymerization the block was trimmed so that the grid was cut through its thickness and the sections on the grid were cut perpendicularly to the original sectioning plane. Electron micrographs of the re-sectioned sections were recorded and the thickness of the sections measured.

Three-dimensional (3D) analysis of the data was made by alignment of the serial sections using OpenCAR software, version 1.5.79 (Sätzler et al., 2002). Afterwards, structures such as presynaptic membrane, AZ, and synaptic vesicles (SVs) were manually contoured. The 3D geometry was reconstructed from the contours of the entire stack of images. Calculations of the volumes of the presynaptic segments, and number and distribution of SVs were done with CARnEval, version 0.10, as previously described (Sätzler et al., 2002). AZ surface area was calculated by multiplying the summed length of the AZs by the thickness of the section (Taschenberger et al., 2002). Membrane regions meeting all of the following three criteria were used for reconstruction of individual AZs: (1) presynaptic and postsynaptic elements are opposed to each other, forming the synaptic cleft, and clearly distinguishable electron-dense material is present on both sides; (2) SVs are located in close proximity to the presynaptic membrane; (3) the structure continues through at least three constitutive sections. SVs located within an arbitrary distance of 400 nm from the AZ are described as “SV cluster” and were included in the analysis. All SVs that were located at distances ≤10 nm from the AZ were classified as “docked SVs.” Distance between the membrane specializations and SVs (SV–AZ distance) was calculated in 2D separately for each section as the perpendicular line from the SV gravity center to the AZ outline, and corrected for SV radius. Nearest-neighbor distance was measured as the distance between the gravity centers of two neighboring objects. SV radius correction was not done for nearest-neighbor analysis. When measuring the SV diameter, it was assumed that the vesicle is a perfect sphere without any membrane irregularities. We did not take account of the vesicle membrane thickness. The vesicle volume (Figure 10) was calculated according to the formula: $\frac{4}{3}\pi r^{3}$, where $r=\frac{\text{SV diameter}}{2}$.

### Statistical analysis

Statistical analysis and curve fitting was performed using Prism 5.0b (GraphPad Software, San Diego California USA). Curve fits used built-in functions, were performed on group mean values unless otherwise indicated, with outlier exclusion and with minimal parameter constraints. Variability arises mostly between individual calyces and to a lesser degree from individual animals (Grande and Wang, 2011). This allowed us to group the measurements performed in different animals. Unequal sample size was due to the complexity of the experimental design. Between-group comparisons were made using one-way ANOVA, followed by Tukey-Kramer *post-hoc* test with significance level *p* < 0.05, or non-parametric Mann Whitney test (non-parametric *t*-test) with significance level *p* < 0.05, unless otherwise indicated. Mean ±s.e.m. values are shown in summary data plots. Photomicrographs were generated with Image J 1.41o, AMIRA 4.1.1 and Adobe Photoshop CS4, version 10.0. Final images were aligned and labeled in Adobe Illustrator CS4, version 13.0.0.

## Results

### Identification and distribution of endogenous synapsin isoforms in the calyx of held

**Figure 1:**
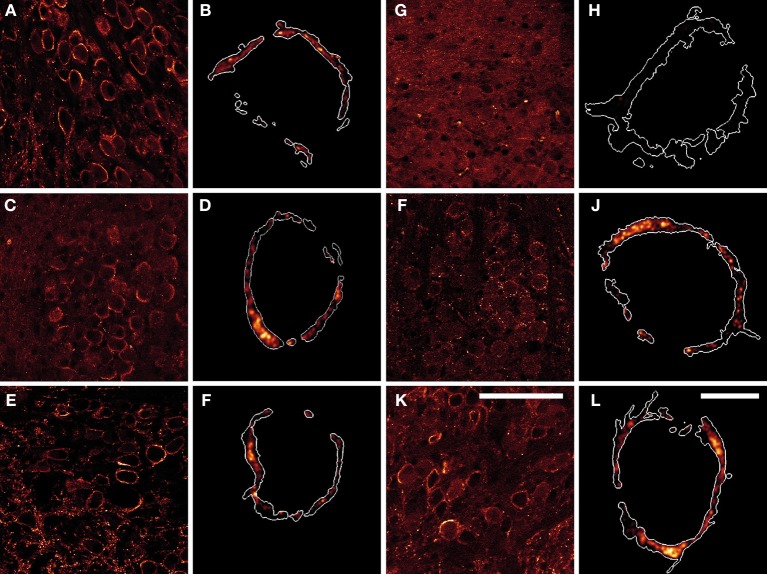
**Products of all three synapsin genes were detected in the calyx of Held, showing similar expression patterns. (A,B)** pan-synapsin I; **(C,D)** synapsin Ib; **(E,F)** pan-synapsin II; **(G,H)** synapsin IIa; **(I,J)** synapsin IIb; **(K,L)** synapsin IIIa. Overview images of the MNTBs **(A,C,E,G,I,K)** stained with synapsin specific antibodies revealed the synapsin isoforms present within the MNTB. High-resolution confocal images **(B,D,F,H,J,L)** allowed the precise localization of synapsin isoforms within the calyx of Held. Expression of mGFP delineates the calyx membrane, and is presented as a white outline in the magnified images. Synapsin isoforms distribute within the entire volume of the calyx of Held, labeled with mGFP. Images represent single confocal planes in pseudocolors. Scale bars: overviews are 100 μm; single calyces (merged-mGFP) are 10 μm.

To identify the synapsin isoforms contributing to synaptic transmission at the rat calyx of Held at P16, we determined the expression and distribution pattern of endogenous synapsins within the presynaptic terminal, using immunohistochemistry (Figure 1). Overview images show that multiple synapsin isoforms were present in the MNTB of P16 rats, where they distributed to the calyx of Held terminals, seen as oval shapes. The overall immunostaining pattern was comparable for the majority of the tested synapsin isoforms–pan-synapsin I (Figures 1A,B), synapsin Ib (Figures 1C,D), pan-synapsin II (Figures 1E,F), synapsin IIb (Figures 1I,J), and synapsin IIIa (Figures 1K,L). Contrary to expectations, synapsin IIa staining was not seen within the MNTB, but only in small terminals outside the MNTB (Figure 1G).

To study the localization of synapsin isoforms inside the presynaptic compartment, calyx of Held terminals were labeled with mGFP protein via stereotaxic delivery of rAAV1/2 particles in the aVCN (Wimmer et al., 2004). Prelabeling with mGFP was necessary to visualize the entire volume of the presynaptic terminals and to exclude numerous non-calyceal terminals contacting the principal cells of the MNTB (Dondzillo et al., 2010). The high-resolution images in Figure 1 depict examples of mGFP-labeled calyces from which the synapsin immunosignal was excised: synapsin I (Figure 1B), synapsin Ib (Figure 1D) synapsin II (Figure 1F), synapsin IIa (Figure 1H), synapsin IIb (Figure 1J), synapsin IIIa (Figure 1L). Although synapsin IIa could be detected in the hippocampus at this age (data not shown), it was absent from the calyx of Held (Figure 1H). With the exception of synapsin IIa, synapsin isoform staining revealed clusters of different sizes within the entire volume of the presynaptic compartment. The regions of maximum intensity were usually detected within the bulk volume of the terminal, distally from the presynaptic membrane, and might represent clusters of SVs away from the AZ. The intensity of the detected immunofluorescent signal was low close to the presynaptic membrane facing the postsynaptic cell, where multiple AZs are located (Sätzler et al., 2002; Dondzillo et al., 2010), coinciding with a lower number of SVs or an absence of synapsin isoforms from these regions (Hirokawa et al., 1989). This result indicates that multiple synapsin isoforms coexist within the calyx of Held.

**Figure 2:**
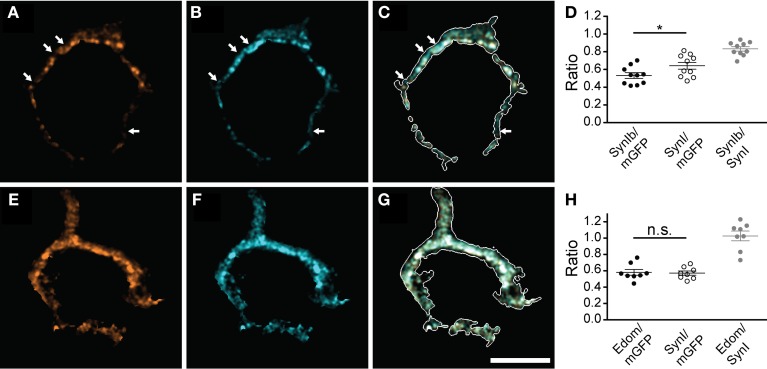
**Multiple synapsin isoforms are co-expressed within the same presynaptic domains.** Calyces stained with isoform-specific antibodies **(A,E)**, and pan-synapsin I antibody **(B,F)**. Overlap with calyx outline, based on the mGFP-expression delineates the membrane of the calyx in the merged images (white outline, **C,G**). The volume of the entire calyx was calculated from mGFP labeling (see Materials and Methods). **(A–C)** synapsin Ib. Arrows indicate pan-synapsin I positive clusters with no detectable synapsin Ib. **(D)** Ratios of the presynaptic volume occupied by synapsin Ib and synapsin I. Synapsin Ib occupied 0.53 ± 0.03, and synapsin I occupied 0.64 ± 0.04 of the presynaptic volume (*N* = 10 presynaptic terminals, Student's *t*-test, *P* = 0.04). Overlap of synapsin Ib and synapsin I signal was 0.83 ± 0.02. **(E–G)** Synapsin E-domain revealing all isoforms containing E-domain (i.e., synapsin Ia, IIa, IIIa). **(H)** Ratios of the presynaptic volume occupied by synapsin E domain and synapsin I. Synapsin E domain occupied 0.58 ± 0.04, synapsin I occupied 0.57 ± 0.03 of the presynaptic volume (*N* = 8 presynaptic terminals, Student's *t*-test, *P* = 0.81). The overlap of synapsin E domain and synapsin I volume was 1.03 ± 0.04. Images represent single confocal planes in pseudocolors. Scale bar is 10 μm. ^*^P < 0.05.

Next, we tested if all synapsin isoforms occupy the same presynaptic volume. For this purpose we examined the localization of specific isoforms with respect to the distribution of synapsin I (Figure 2). Synapsin Ib immunofluorescence revealed a more restricted, punctate pattern (Figure 2A), while pan-synapsin I staining was widely distributed, filling up a larger fraction of the presynaptic volume (Figure 2B). Several spots were positive only for pan-synapsin I, but negative for synapsin Ib (Figures 2A–C arrows), suggesting that they contain only synapsin Ia, assuming similar labeling efficiencies. The difference between presynaptic volume occupied by synapsin Ib vs. synapsin I was small, but significant (Figure 2D, Student's *t*-test, *P* = 0.04). Staining with an antibody recognizing the E-domain specifically visualizes the a-isoforms (Figures 2E–G). Because synapsin IIa is not expressed in the calyx, this antibody reports location of synapsin Ia and IIIa. Extensive overlap of the E-domain signal with the pan-synapsin I signal suggests that synapsin IIIa is present in the same volume as synapsin I (Figure 2H, Student's *t*-test, *P* = 0.81). In summary, synapsin Ia, Ib, IIb and IIIa are endogenously expressed in the rat calyx of Held at P16 and are co-expressed within the same volume of the presynaptic terminal, apart from a possible subcompartmentalization of synapsin Ia and Ib expression. In contrast, the synapsin IIa isoform is not present in appreciable quantity in the calyx of Held.

### Colocalization of synapsin isoforms with synaptic vesicles

**Figure 3:**
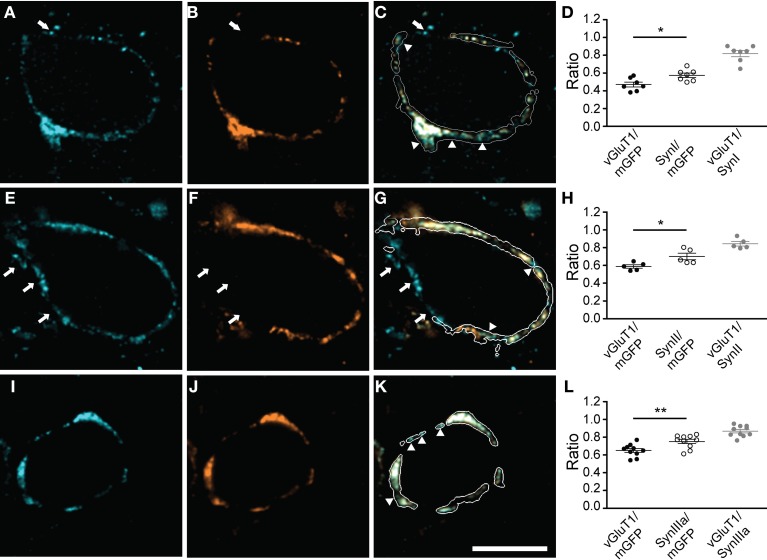
**All synapsin isoforms colocalize with synaptic vesicles within the calyx of Held.** The volume of the entire calyx was calculated from mGFP labeling (see Materials and Methods). Distribution of synapsin isoforms **(A,E,I)** compared to vGluT1, a SV marker **(B,F,J)**, overlay of the corresponding image planes within the calyx outline **(C,G,K)**. Arrowheads indicate synapsin signal outside the vesicle cluster. White arrows highlight synapsin-positive clusters outside the mGFP-outlined presynaptic membrane, which do not show vGluT1 positive immunofluorescence. **(A–C)** pan-synapsin I; **(E–G)** pan-synapsin II; **(I–K)** synapsin IIIa. Ratios of the presynaptic volume occupied by vGluT1 and synapsin isoforms. **(D)** Synapsin I occupied 0.58 ± 0.03, vGluT1 occupied 0.47 ± 0.02 of the presynaptic volume (*N* = 7 presynaptic terminals, Student's *t*-test, *P* = 0.02). The overlap of vGluT1 and synapsin I was 0.82 ± 0.03. **(H)** Synapsin II occupied 0.7 ± 0.02, vGluT1 occupied 0.58 ± 0.04 of the presynaptic volume. (*N* = 5 presynaptic terminals, Student's *t*-test, *P* = 0.03). **(L)** The overlap of vGluT1 and synapsin II was 0.84 ± 0.03. Synapsin IIIa occupied 0.75 ± 0.02, vGluT1 occupied 0.65 ± 0.02 of the presynaptic volume (*N* = 10 presynaptic terminals, Student's *t*-test, *P* = 0.01). The overlap of vGluT1 and synapsin IIIa volume was 0.87 ± 0.02. Images represent single confocal planes in pseudocolors. Scale bar is 10 μm. ^*^P < 0.05; ^**^P < 0.01.

Since synapsins cluster SVs but can also exist in an unbound cytosolic state (Cesca et al., 2010; Shupliakov et al., 2011), we correlated synapsin signal with SVs distribution. SVs were visualized using an antibody directed against vGluT1. All synapsin isoforms expressed in the calyx of Held colocalized with the vGluT1 immunosignal within the boundaries of the presynaptic membrane (Figure 3, and data not shown). While the overlap with SVs was extensive for all synapsin isoforms, in each of the examples shown in Figures 3C,G,K (arrowheads) synapsin signal occupied a larger volume of the calyx than SVs (Figures 3D,H,L, Student's *t*-test *P* = 0.02, 0.03, 0.01, respectively). Although the calyx of Held expresses both vGluT1 and vGluT2, their expression patterns are largely overlapping at the age examined here (Billups et al., 2005; Blaesse et al., 2005). Hence, the areas devoid of vGluT1 may reflect synapsin molecules not bound to SVs; however, we cannot exclude that some vGluT2-positive vesicles may not be visualized.

Synapsin-positive signals could also be detected within putative extracalyceal terminals onto the principal cell of the MNTB; however, outside the calyx terminal synapsin-positive puncta failed to contain vGluT1 (Figure 3, arrows). In 64 out of 75 synapses examined vGluT1 immunoreactivity was restricted to the presynaptic volume outlined by mGFP, making this presynaptic protein a specific, but not exclusive, marker for SVs in the calyx of Held. Synapsin IIIa was primarily restricted to the calyx of Held. In contrast, synapsin I and II isoforms were not restricted to the calyx of Held and were present in extracalyceal synapses, which may be inhibitory, contacting the surface of the principal cell (Lenn and Reese, 1966; Nakajima, 1971; Awatramani et al., 2005). Although the evidence for non-calyceal somatic excitatory inputs is equivocal at this age, synapsin positive puncta outside the calyx boundaries may also represent small excitatory synapses (Morest, 1968; Guinan and Li, 1990; Hamann et al., 2003), utilizing glutamate transporters different than vGluT1 (Blaesse et al., 2005)

### Proper targeting of overexpressed synapsin Ia/Ib and redistribution of SVs within perturbed terminals

**Figure 4:**
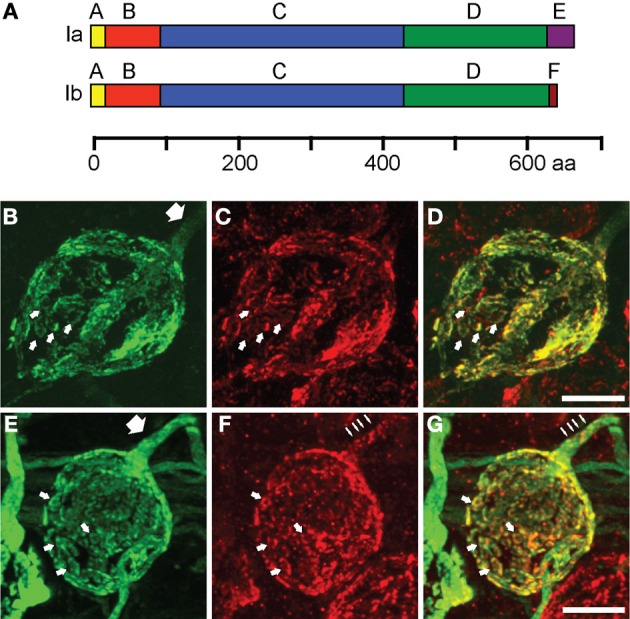
**EYFP-synapsin I fusion proteins are successfully expressed in the calyx of Held upon rAAV-mediated infection. (A)** Domain structure of synapsin Ia and synapsin Ib. The two isoforms share structural homology with varying C-termini. The longer E-domain in synapsin Ia, implicated in binding to SV membrane, is exchanged for the F-domain in synapsin Ib. **(B)** AAV1/2 mediated expression of synapsin Ia (green) in the calyx of Held 10 days after stereotaxic delivery of rAAV into the aVCN. **(C)** Staining of vGluT1 (red) revealed SV clusters. **(D)** Overlay of synapsin Ia and SV. **(E–G)** Same as **(B–D)**, but for overexpression of EYFP-synapsin Ib. White arrows indicate SV donuts, thick arrows point out volume-filling expression pattern of EYFP-synapsins in calyceal axons, thin arrows indicate clustered distribution of vGluT1 within the axon. Images represent entire confocal stacks collapsed in the z-dimension. Scale bars are 10 μm.

To study how single isoforms maintain synaptic transmission at the calyx of Held, we perturbed the molecular composition of the presynaptic terminal by infecting the aVCN with rAAV1/2 particles coding for either synapsin Ia or synapsin Ib, N-terminally fused to EYFP for visualization (Figure 4A). Ten days after stereotaxic injection, both proteins successfully targeted to the presynaptic terminal, where they filled most of the presynaptic volume and colocalized with vGluT1, similar to the endogenous isoforms (Figures 4B,E). vGluT1-positive puncta were detected throughout the entire structure in both cases (Figures 4C,F). vGluT1 signal was a subset of the EYFP-synapsin signal (Figures 4D,G), consistent with an excess of synapsin molecules invading the synapse due to overexpression, part of which remained unbound to vesicles (Cesca et al., 2010; Shupliakov et al., 2011). Examination of the axonal segment leading into the calyx and the palm region of the calyx revealed the presence of both EYFP-synapsin Ia (Figure 4B, white arrow) and EYFP-synapsin Ib (Figure 4E, white arrow). The distribution of overexpressed synapsin isoforms in the calyceal axon (Figures 4B,E) as well as of endogenous synapsin isoforms (Figures 2E–G) contrasts with the discrete localization of small vGluT1 positive clusters in the axons (Figures 4F,G thin arrows). Both overexpressed proteins were distributed within individual stalks along the circumference of the postsynaptic cell and within structural specializations of the synapse (small arrows), which are composed of SVs organized around clusters of mitochondria (Wimmer et al., 2006).

**Figure 5:**
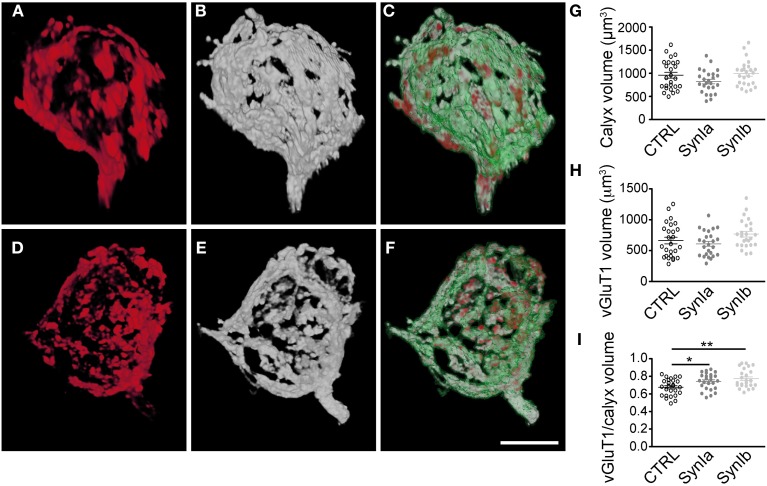
**Overexpression of synapsin I isoforms leads to redistribution of SVs within the calyx of Held.** 3D reconstruction of calyces overexpressing mGFP (green outline) together with synapsin I isoforms (white), stained for vGluT1 (red). **(A–C)** Overexpression of EYFP-synapsin Ia. **(D–E)** Overexpression of EYFP-synapsin Ib. Merged images (**C** and **E**) represent overlay of overexpressed mGFP, synapsin isoform and vGluT1. **(G–I)** Quantitative analyses for control, synapsin Ia and synapsin Ib overexpression. **(G)** Volume of the space taken up by the mGFP-labeled presynaptic terminal. **(H)** Volume taken up by SVs, stained for vGluT1. **(I)** The Ratio of SV volume to calyx volume is increased by the overexpression of both synapsin I isoforms (One-Way ANOVA, *p* = 0.002). *N* = 26 CTRL calyces from 5 animals, 26 EYFP-synapsin Ia overexpressing calyces from 4 animals, 25 EYFP-synapsin Ib overexpressing terminals from 5 animals. Open black symbols represent control, light gray symbols represent EYFP-synapsin Ia, open gray symbols represent EYFP-synapsin Ib. Scale bar is 10 μm. ^*^P < 0.05; ^**^P < 0.01.

To study the effect of synapsin overexpression on SV distribution within the calyx of Held, we performed double infections with rAAV1/2 coding for mGFP, and rAAV1/2 coding for the synapsin isoform of interest (Figure 5). SV distribution was examined by staining with vGluT1 antibody followed by 3D structural analysis of the entire presynaptic compartment. As a control, the distribution of vGluT1 was examined in terminals expressing mGFP alone (data not shown). We examined the 3D distribution of SV clusters (Figures 5A,D) with respect to synapsin I isoforms (Figures 5B,E) within the mGFP delineated calyx boundaries (Figures 5C,F). It is of note that both the signal strength (amount of protein, affinity of the antibodies, efficiency of detection) and spatial distribution of the signals contribute to the measured presynaptic volume of both vGluT1 and synapsin isoforms. 3D-analysis showed that the general architecture and the volume of the calyx of Held remained unaltered in comparison to controls (Figure 5G, 961 ± 63 μm^3^ for control calyces, 803 ± 51 μm^3^ for synapsin Ia and 995 ± 55 μm^3^ for synapsin Ib overexpressing calyces; one-way ANOVA, *P* = 0.044; Tukey-Kramer *post-hoc* test did not reveal significant differences between groups). Quantification of the vGluT1 immuno-signal did not reveal an effect of synapsin Ia or synapsin Ib overexpression on the level of vGluT1 (Figure 5H), at odds with previous observations that loss of synapsins reduced the expression level of presynaptic proteins (Gitler et al., 2004b; Bogen et al., 2009; Vasileva et al., 2012). The volume occupied by SVs, visualized via vGluT1 immunofluorescence was unaffected: 663 ± 54 μm^3^ for control synapses, 596 ± 41 μm^3^ for synapsin Ia, and 760 ± 47 μm^3^ for synapsin Ib overexpressing terminals (one-way ANOVA, *P* = 0.06; Figure 5H). Because the size of the presynaptic terminals can vary substantially even within the same preparation (Grande and Wang, 2011), we calculated the ratio of the volume occupied by vGluT1 immunofluorescence to the volume of the entire calyx of Held. Overexpression of synapsin I isoforms shifted this ratio to significantly higher values of 0.74 ± 0.02 for synapsin Ia and 0.77 ± 0.02 for synapsin Ib-overexpressing calyces vs. 0.68 ± 0.02 for control calyces (one-way ANOVA, *P* = 0.002; Figure 5I). Thus, the same total amount of SVs occupied a larger part of the presynaptic volume compared to control terminals. Therefore, we conclude that overexpression of either synapsin I isoform at the calyx of Held leads to vesicle redistribution within the volume of the calyx of Held.

### Synapsin I isoforms do not control basal transmission at the calyx of held

Pronounced redistribution of vesicles within the calyx of Held implies functional abnormalities due to overexpression of synapsin I isoforms. To analyze putative alterations in the calyx of Held function we performed whole-cell voltage clamp recordings from MNTB principal cells associated with infected presynaptic terminals. Spontaneous quantal excitatory postsynaptic events (spEPSCs) were recorded in MNTB principal cells for 2–4 min after establishing the whole-cell configuration, and prior to any high-frequency stimulation. The properties of spEPSC (charge, frequency, and kinetics) were not changed by the overexpression of synapsin Ia or synapsin Ib (Table 1). Hence, quantal size was unaffected by overexpression of either of the synapsin isoforms tested, consistent with results obtained from triple knock-out calyces (Vasileva et al., 2012). Neither synapsin Ia nor Ib overexpression affected rise time or decay kinetics of quantal events.

**Table 1:** **Synaptic parameters for wild type and synapsin Ia or synapsin Ib overexpressing calyces**.

**Basal transmission**				
**spESPC PROPERTIES**				
	**Frequency (Hz)**	**Charge (pC)**	**Rise time (μs)**	**Decay time (μs)**
CTRL	1.7 ± 0.2 (16)	31.8 ± 1.5 (3238 events)	110.3 ± 4.7	352 ± 24
EYFP-synIa	2.1 ± 0.4 (21)	29.1 ± 1.2 (7943 events)	113.9 ± 2.4	356 ± 14
EYFP-synIb	1.9 ± 0.4 (19)	31.9 ± 1.9 (3478 events)	109.2 ± 3.3	349 ± 17
One-way ANOVA	*P* = 0.68	*P* = 0.32	*P* = 0.56	*P* = 0.96
**eESPC PROPERTIES**				
	**eEPSC size (nA)^a^**	**Quantal content**	**RRP size (SVs)^b^**	**Pr (EPSC_1_/RRP)^b^**
CTRL	18.4 ± 2.3 (36)	259 ± 20 (16)	1664 ± 188 (12)	0.17 ± 0.01 (12)
EYFP-synIa	14.3 ± 1.3 (21)	291 ± 35 (12)	1792 ± 226 (5)	0.28 ± 0.02 (15)^**^
EYFP-synIb	15.5 ± 1.9 (27)	322 ± 29 (15)	1578 ± 109 (4)	0.23 ± 0.02 (11)
One-way ANOVA	*P* = 0.37	*P* = 0.25	*P* = 0.85	*P* = 0.005

Number in parentheses indicates number of replicates per condition.

a EPSC size is estimated by extrapolation at V_h_ = −70 mV.

b RRP and Pr estimated from 300 Hz depression train.

** P < 0.01.

Evoked EPSCs (eEPSCs) were measured in response to afferent fiber stimulation at 0.1 Hz, evoking an all-or-none response in the MNTB principal cells. Basal eEPSC magnitude (see Materials and Methods) did not significantly differ between control and synapsin overexpression conditions (Table 1). The number of SVs released per action potential-like stimulus (quantal content) was also not altered after synapsin overexpression (Table 1).

### Synapsin I isoforms control use-dependent synaptic plasticity at the calyx of held

**Figure 6:**
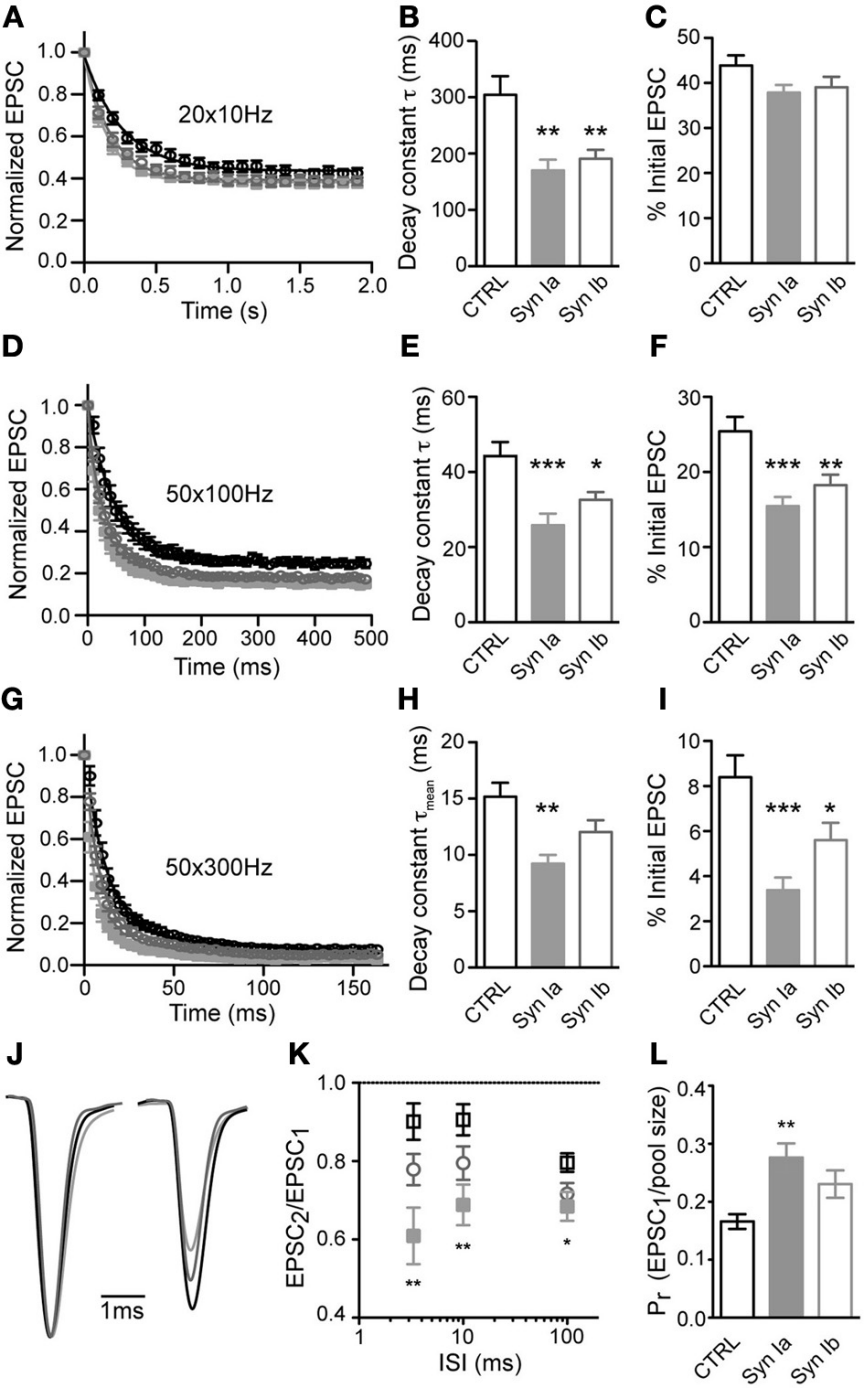
**Overexpression of synapsin Ia or synapsin Ib increases depression during repetitive stimulation.** Synapses were challenged with repetitive stimulation (20–50 pulses) at varying frequency. **(A–C)** Stimulation at 10 Hz. *N* = 20 CTRL calyces from 12 animals, 13 EYFP-synapsin Ia positive terminals from 4 animals, 16 EYFP-synapsin Ib overexpressing calyces from 9 animals. **(D–F)** Depression at 100 Hz. *N* = 20 CTRL calyces from 15 animals, 21 EYFP-synapsin Ia positive terminals from 6 animals, 19 EYFP-synapsin Ib overexpressing calyces from 9 animals. **(G–I)** Depression at 300 Hz. *N* = 17 CTRL calyces from 12 animals, 11 EYFP-synapsin Ia positive terminals from 4 animals, 15 EYFP-synapsin Ib overexpressing calyces from 9 animals. **(J)** Paired-pulse ratio of the first two stimuli in a 300 Hz stimulation train. Traces show an ensemble of recordings from calyces of the three genotypes studied. Sweeps were taken at −30 mV holding potential, and normalized and aligned to the first peak. Stimulation artifacts have been removed for clarity. **(K)** Paired-pulse ratio (PPR) for the three genotypes, across the range of frequencies studied. **(L)** Calculated release probability of calyx of Held terminals overexpressing synapsin I isoforms compared to CTRL measurements. Open black symbols represent control, light gray symbols represent EYFP-synapsin Ia, open gray symbols represent EYFP-synapsin Ib. ^*^P < 0.05; ^**^P < 0.01; ^***^P < 0.001.

Trains of repetitive stimulation were used to analyze the properties of synaptic depression at the calyx of Held. At 10 Hz stimulation frequency, all three conditions showed similar depression that reached a steady-state after ~ 10 stimuli (Figure 6A). Onset of depression, fit by a monoexponential function per cell, had a time constant (τ) of 304 ± 33 ms in control cells, and was significantly faster in synapses overexpressing synapsin Ia (170 ± 19 ms) or synapsin Ib (191 ± 16 ms; one-way ANOVA, *P* < 0.01; Figure 6B). However, the steady state level of depression (average of 10th–20th stimuli, determined individually for each cell) was similar for all three conditions: control synapses showed steady-state depression of 43.9 ± 2.2% of the initial EPSC magnitude, whereas synapsin Ia positive terminals depressed to 37.9 ± 1.7% and synapsin Ib–to 39.1 ± 2.3%, of the initial EPSC magnitude (one-way ANOVA, *P* = 0.12; Figure 6C).

Stimulation at higher frequency (100 Hz) resulted in strong depression, which was fit by a monoexponential function for responses in individual cells from the 2–50th stimuli (Figure 6D). The first stimulus was excluded because of occasional facilitation in the EPSC. Control synapses depressed with a time constant of 44.2 ± 3.7 ms to 25.4 ± 1.9 % of the initial state. The rate and extent of depression was significantly and similarly altered by overexpression of either synapsin Ia or synapsin Ib, leading to faster depression and a smaller steady-state level of transmission (Figures 6E,F). Synapsin Ia overexpression resulted in depression with a time constant of 26.4 ± 3.0 ms, and steady state decreased to 15.4 ± 1.2% (one-way ANOVA, *P* < 0.001 for both indices). Synapsin Ib overexpression likewise sped depression, with a time constant of 32.6 ± 2.1 ms (one-way ANOVA, *P* < 0.01), and a steady state of 18.2 ± 1.4% (one-way ANOVA, *P* < 0.05). Thus, overexpression of either synapsin Ia or synapsin Ib resulted in faster and more severe depression at 100 Hz compared to control terminals.

Additionally, we stimulated control and infected terminals at 300 Hz, which can be regarded as the upper bound on stimulation frequency at room temperature (Taschenberger and von Gersdorff, 2000; Renden et al., 2005), and monitored synaptic responses from stimuli 2–50 (Figure 6G). Notably, at this frequency synaptic depression was not monoexponential, but consisted of an additional fast (<10 ms) phase in both control and genetically manipulated synapses. The time constants (τ and relative weight for τ_fast_) of short-term depression in control synapses were τ_fast_ = 7.4 ± 1.0 ms (70.0 ± 1.3%), and τ_slow_ = 33.5 ± 2.8 ms. Overexpression synapsin Ia resulted in significantly faster τ_fast_ (4.0 ± 0.5 ms; one-way ANOVA, *P* < 0.05) and τ_slow_ (21.64 ± 2.54 ms; one-way ANOVA, *P* < 0.05) but a similar relative weight (68.0 ± 2.3%). The mean depression time constant (τ_mean_) was calculated according to the formula: [τ_mean_ = τ^*^_fast_(%τ_fast_) + τ^*^_slow_(100%–%τ_fast_)]. τ_mean_ for control synapses was 15.2 ± 1.2 ms. Synapsin Ia overexpression resulted in a significantly faster mean depression time constant (τ_mean_ = 9.2 ± 0.8 ms, one-way ANOVA, *P* < 0.01). Notably, overexpression of synapsin Ib did not lead to acceleration of depression—τ_fast_ was 6.01 ± 0.66 ms with relative weight 73.3 ± 1.7%, and τ_slow_ of 31.1 ± 3.0 ms. This resulted in τ_mean_ of 12.7 ± 1.0 ms (one-way ANOVA, *P* > 0.05; Figure 6H). Control synapses depressed to 8.4 ± 1.0% of the initial EPSC magnitude by the end of the 300 Hz train. This steady-state was significantly reduced to 3.4 ± 0.6% by synapsin Ia (one-way ANOVA, *P* < 0.001) and to 5.6 ± 0.8% by synapsin Ib overexpression (one-way ANOVA, *P* < 0.05; Figure 6I).

Using the data presented above, we examined the PPR for potential differences in facilitation or depression early in the stimulus train, due to synapsin I overexpression (Figures 6J,K). Consistent with an increase in PPR in hippocampus of synapsin I knock-out mice (Rosahl et al., 1993), synapsin Ia overexpression significantly affected PPR at all of the interstimulus intervals (ISI) tested (one-way ANOVA, *P* < 0.01 for ISI 3.33 ms, 10 ms, and *P* < 0.05 for ISI 100 ms). There was, however, no effect of synapsin Ib overexpression on the PPR at any ISI.

An extrapolation of the data provided by depression trains at 100 Hz allows an estimation of the size of the RRP (Schneggenburger et al., 1999; Stevens and Williams, 2007). According to this estimation, the RRP contained 1664 ± 188 SV in control terminals, consistent with previous reports (Iwasaki and Takahashi, 2001; Kushmerick et al., 2006). Overexpression of neither synapsin I isoform significantly changed the size of the RRP (Table 1). Using this measurement, we observed a highly significant increase in the *P*_*r*_ of synapses overexpressing synapsin Ia, but not synapsin Ib, relative to controls (Figure 6L; Table 1). This result is consistent with those from PPR and depression experiments indicating an acceleration of SV release following activity. Thus, overexpression of synapsin I isoforms accelerated short-term depression at the calyx of Held without altering the properties of the readily-releasable SVs.

### Recovery from depression is accelerated in calyces overexpressing synapsin Ia

**Figure 7:**
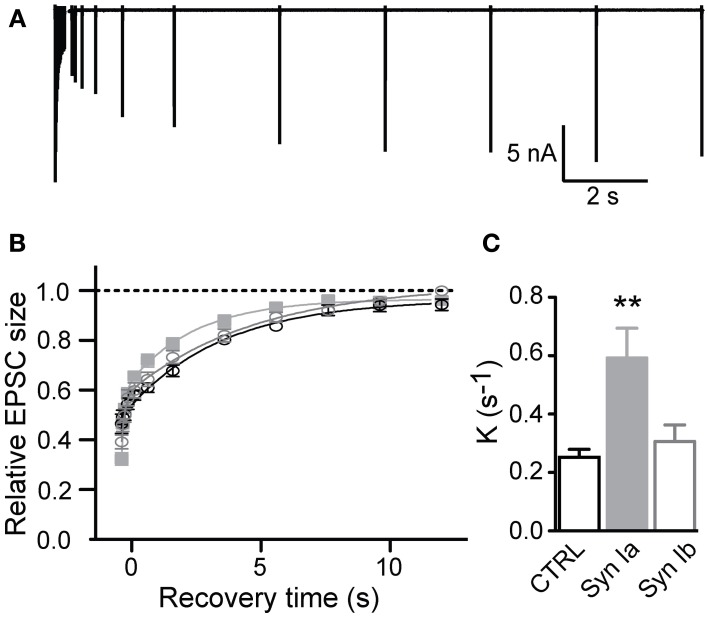
**Synapsin Ia accelerates recovery from depression after strong stimulus trains. (A)** Sample trace, illustrating the recovery from depression stimulation protocol. Synapses were depressed with a stimulation train and recovery of EPSC amplitudes was measured at varying intervals. **(B,C)** Recovery from a 20 × 100 Hz depression train. Due to the variance in the steady state of STD between genotypes (see Figure 6), depression level was set to zero and recovery kinetics were measured from the resulting curves. **(B)** Peak amplitudes are shown relative to the initial EPSC amplitude. **(C)** Recovery rate was significantly faster in synapses overexpressing synapsin Ia. For simplicity monoexponential rates of recovery were plotted, see text for details. *N* = 8 CTRL from 6 animals, *N* = 7 EYFP-synapsin Ia from 5 animals, *N* = 9 EYFP-synapsin Ib overexpressing calyces from 6 animals. Open black symbols represent control, light gray symbols represent EYFP-synapsin Ia, open gray symbols represent EYFP-synapsin Ib. ^**^P < 0.01.

Following short trains at 100 Hz, recovery of the partially-depleted pool of release-competent vesicles was assayed, using single test pulses at variable intervals following the end of the stimulus train (varying from 64 ms to 12 s rest intervals; Figure 7A). Recovery was calculated as (EPSC_*n*_–EPSC_*ss*_)/(EPSC_1_-EPSC_*ss*_), where EPSC_*n*_ represents the measured EPSC after the depleting train, EPSC_*ss*_ is the steady-state EPSC integral, to account for different levels of depression between the control and infected terminals, and EPSC_1_ is the first response in the depletion train.

For a short train (6 pulses at 100 Hz), transmission was depressed to ~ 50% in control synapses, and recovered in all conditions with a monoexponential time course (*K* = 0.22 ± 0.02 s^−1^, *r*^2^ > 0.996 in control condition). Overexpression of either synapsin Ia or Ib did not significantly affect the time course of recovery in this protocol, with synapses overexpressing synapsin Ia recovering at a rate *K* = 0.23 ± 0.01 s^−1^, and synapsin Ib-overexpressing synapses recovering at *K* = 0.22 ± 0.02 s^−1^(one-way ANOVA, *P* = 0.86).

Synaptic recovery after a longer depleting train (20 pulses at 100 Hz; Figures 7B,C) was complete within 10–12 s as reported previously (Kushmerick et al., 2006). In control terminals recovery followed a monoexponential time course with rate constant *K* = 0.25 ± 0.03 s^−1^ (*r*^2^ > 0.995); however, overexpression of each synapsin I isoform resulted in significantly faster recovery to the initial EPSC magnitude. It is evident that synapsin Ia overexpression significantly speeds the recovery of EPSCs (Figure 7B). On further examination, this recovery proved to be the result of the introduction of an additional fast kinetic component in the infected synapses, resulting in a better fit of the recovery data by a biexponential function in synapsin Ia and synapsin Ib overexpressing synapses (*P* < 0.001; extra sum of squares *F* test; *r*^2^ > 0.997). Synapsin Ia overexpression sped recovery due to an increase in the weight of the fast component (*K*_fast_ = 6.83 ± 1.58 s^−1^), to 37.7 ± 2.7% of recovery (*P* = 0.05 vs. synapsin Ib, Student's *t*-test), and a (*K*_slow_ = 0.36 ± 0.04 s^−1^). Recovery in synapsin Ib-expressing terminals resulted in *K*_fast_ = 7.19 ± 2.73 s^−1^ (contributing 28.14 ± 3.4 % of total recovery) and *K*_*slow*_ = 0.20 ± 0.03 s^−1^, which was significantly slower than the synapsin Ia slow component (*P* = 0.003, Student's *t*-test). By contrast, control data could not be fit double-exponentially without constraints. To be able to make a direct comparison with the control the data from the infected terminals was fit monoexponentially with rate constants of *K* = 0.25 ± 0.03 s^−1^ for control terminals and *K* = 0.59 ± 0.1 s^−1^ for synapsin Ia and *K* = 0.31 ± 0.06 s^−1^ for synapsin Ib overexpressing calyces (one-way ANOVA, *P* < 0.01; Figure 7C).

From these results, we conclude that overexpression of either synapsin I isoform speeds synaptic depression due to SV depletion with a significant action of synapsin Ia on activity-dependent release probability. Furthermore, our results indicate that synapsin Ia acted specifically to augment the fast component of synaptic recovery following partial depression of the RRP.

### Ultrastructural alterations at the calyx of held upon overexpression of synapsin Ia

**Figure 8:**
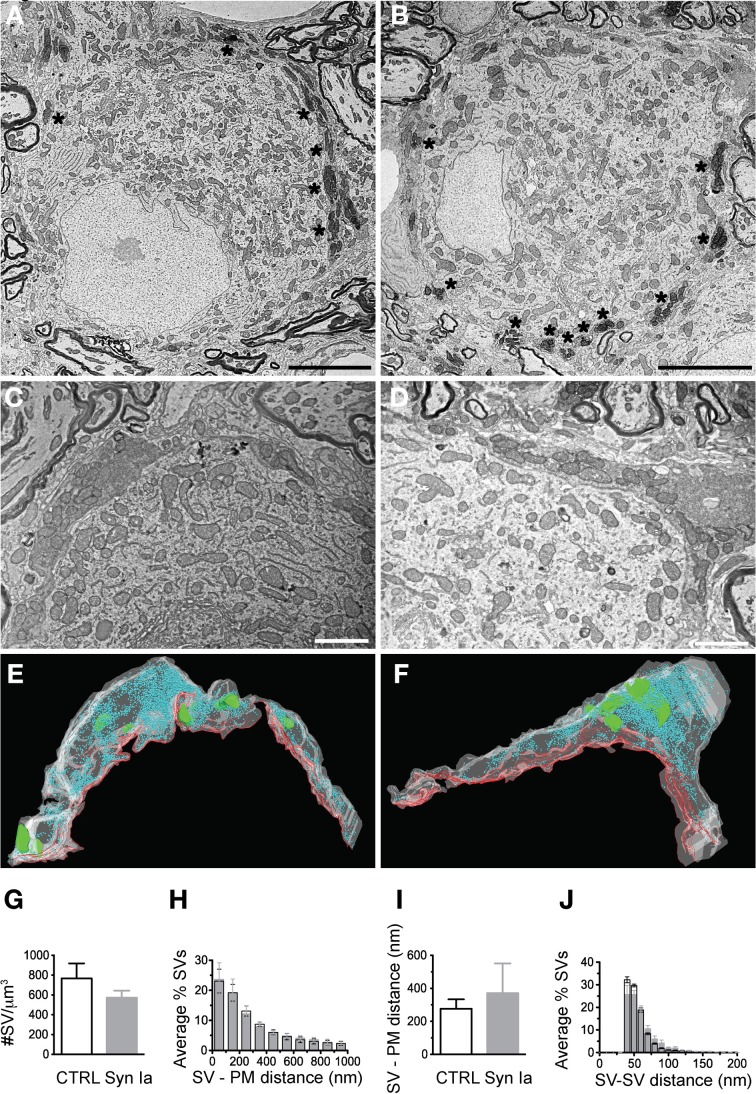
**Photooxidation and S^3^EM allowed the ultrastructural identification of genetically perturbed calyces.** Low magnification images of calyces expressing EGFP **(A)** or EGFP-synapsin Ia **(B)** showing characteristic electron-dense material along the perimeter of the MNTB principal cell allowed identification of infected terminals. High magnification images of EGFP **(C)** and EGFP-synapsin Ia **(D)**. **(E)** 3D reconstructions of the calyceal segment shown in **(C)**. **(F)** 3D reconstruction of the segment shown in **(D)**. SVs are presented as cyan spheres, the presynaptic membrane opposing the MNTB principal cell is shown as red line, the volume of the reconstructed segments is presented in white and is rendered transparent for clarity, parts of the postsynaptic cell engulfed by the presynaptic element are presented in green. **(G)** SV density in the reconstructed segments. **(H)** Histogram of the SV distribution from the presynaptic membrane (PM) opposing the principal cell. **(I)** Median distance of SVs to the presynaptic release face. **(J)** Histogram of the distribution of the nearest distance between neighboring SVs. Open black symbols represent CTRL, light gray symbols represent EGFP-synapsin Ia. *N* = 4 CTRL segments from 2 animals, and *N* = 4 EGFP-synapsin Ia segments from 2 animals. Scale bars: **(A)** and **(B)** 5 μm; **(C)** and **(D)** 1 μm.

Overexpression of synapsin Ia resulted in a robust functional phenotype, therefore we examined its effect on the ultrastructure of the presynaptic terminal. Photo-oxidation allowed us to identify calyces expressing EGFP or EGFP-synapsin Ia by EM, thereby linking protein expression to the ultrastructural phenotype (Horstmann et al., 2013). In both cases, photo-oxidation resulted in the formation of electron-dense material within the calyces, visible at low magnification (Figures 8A,B, asterisks). We selected calyceal segments according to the presence of the electron-dense material, and performed serial-section scanning EM (S^3^EM; (Horstmann et al., 2012). This allowed us to generate 3D reconstructions and examine SV distribution in calyces expressing EGFP or EGFP-synapsin Ia. High-magnification images (Figures 8C,D) show segments from infected presynaptic terminals that were reconstructed (Figures 8E,F). We examined four EGFP-positive and four EGFP-synapsin Ia-positive terminals from two rats each, and reconstructed a total volume of 53.16 μm^3^ from EGFP-positive calyces and 25.44 μm^3^ from EGFP-synapsin Ia-containing terminals. The overall ultrastructure was not affected by the overexpression of synapsin Ia, and the terminals contained comparable SV numbers (Figure 8G; Table 2). To assess alterations in the SV distribution within the terminal, we measured the distance from the SVs to the presynaptic membrane opposing the MNTB principal cell, as well as the distance between adjacent SVs. SVs were present within the entire reconstructed segments and genetic manipulation did not alter the distance from SVs to the plasma membrane, or between the centers of two neighboring SVs (Figures 8H,I; Table 2).

**Table 2:** **Comparison of SV density, distance from presynaptic membrane opposing the MNTB principal cell to SVs and nearest neighbor distance between SVs in all examined synapses overexpressing EGFP and EGFP-synapsin Ia**.

**EGFP**	**Volume (μm^3^)**	**SV #**	**SV/μm^3^**	**PM-SV distance (nm)**	**SV-SV distance (nm)**
Calyx-1	4.08	4171	1022.30	168.20	50.38
Calyx-2	22.73	12236	538.32	420.56	51.51
Calyx-3	17.64	8426	477.66	204.75	51.58
Calyx-4	8.71	9001	1033.41	312.43	49.49
Average	13.3 ± 4.2	8458.5 ± 1657	767.9 ± 150.6	276.5 ± 57	50.7 ± 0.5
**EGFP-syn Ia**	**Volume μm^3^**	**SV #**	**SV/μm^3^**	**PM-SV distance (nm)**	**SV-SV distance (nm)**
Calyx-1	8.95	6547	731.51	910.25	53.73
Calyx-2	1.66	817	492.17	177.51	60.25
Calyx-3	6.70	4330	646.27	162.38	48.98
Calyx-4	8.13	3425	421.28	234.15	56.42
Average	6.4 ± 1.6	3779.8 ± 1185.5	572.8 ± 70.7	371.1 ± 180.4	54.9 ± 2.4
Mann-Whitney test	*P* = 0.34	*P* = 0.34	*P* = 0.49	*P* = 0.89	*P* = 0.34

**Figure 9:**
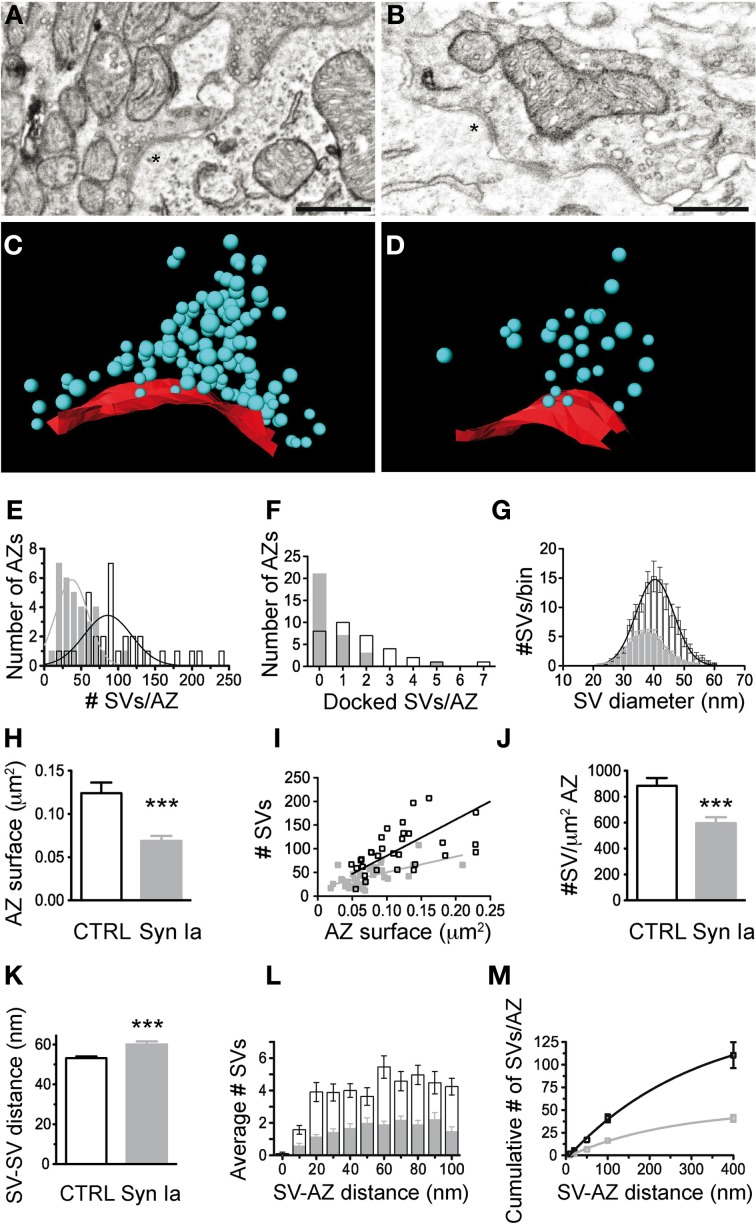
**Synapsin Ia overexpression alters the architecture of the AZs and the proximal vesicle cluster. (A)** Two AZs from a calyx overexpressing EGFP. **(B)** Two AZs from a calyx overexpressing EGFP-synapsin Ia. **(C)** 3D reconstruction of the AZ, highlighted with asterisks in **(A)**. **(D)** 3D reconstruction of the AZ highlighted with asterisks in **(B)**. SVs are presented as cyan spheres, the surface of the AZ is presented in red. **(E)** Number of SVs per AZ. **(F)** Number of docked SVs per AZ. **(G)** Histogram of the distribution of the SV diameter. **(H)** AZ surface area was decreased by the overexpression of synapsin Ia. **(I)** Number of SVs plotted vs. the surface area of the AZ. **(J)** Density of the SVs within the cluster associated with AZs is reduced upon overexpression of synapsin Ia. **(K)** Increased median distance between neighboring SVs upon synapsin Ia overexpression. **(L)** Histogram of the SV distribution within the first 100 nm from the AZ. **(M)** Cumulative distribution of SVs within 400 nm from the AZ. *N* = 33 CTRL AZs from 4 calyces from 2 animals and 32 EGFP-synapsin Ia AZs from 4 calyces from 2 animals. Open black symbols represent CTRL, light gray symbols represent EGFP-synapsin Ia. Scale bars are 200 nm. ^***^P < 0.001.

We next examined the SV distribution in close proximity to the release sites. We traced 33 AZs in EGFP-overexpressing calyces and 32 AZs in EGFP-synapsin Ia-overexpressing terminals. AZs of both EGFP and EGFP-synapsin Ia-overexpressing calyces had associated SV clusters (Figures 9A,B). We performed 3D reconstructions of the AZs (Figures 9C,D) and characterized the SV distribution within the clusters (Figures 9E–M; cf. Materials and Methods). The clusters in control terminals contained 101.3 ± 9.2 SVs per AZ, whereas there were only 41.7 ± 3.9 SVs per AZ due to synapsin Ia overexpression (Mann Whitney test, *P* < 0.0001; Figure 9E). Concomitant to a decrease in the total number of SVs, the vesicles physically attached to the AZ were also significantly decreased (Figure 9F). Although the coefficients of variation were large (C_*v*_EGFP = 0.96 and C_*v*_EGFP-synapsin Ia = 1.86), we counted 1.7 ± 0.3 docked SVs per AZ in control terminals and only 0.6 ± 0.2 docked SVs per AZ in synapsin Ia-overexpressing calyces (Mann Whitney test, *P* = 0.0004). SVs in synapsin Ia-overexpressing terminals were also individually smaller, having an average diameter of 37.57 ± 0.33 nm, while the SVs in control terminals had diameters of 40.2 ± 0.38 nm (Mann Whitney test, *P* < 0.0001; Figure 9G). This resulted in an average vesicle volume of 2.8^*^10^−5^ μm^3^ in synapsin Ia-overexpressing and 3.4^*^10^−5^ μm^3^ in control synapses, which were significantly different (Mann Whitney test, *P* < 0.0001).

**Figure 10:**
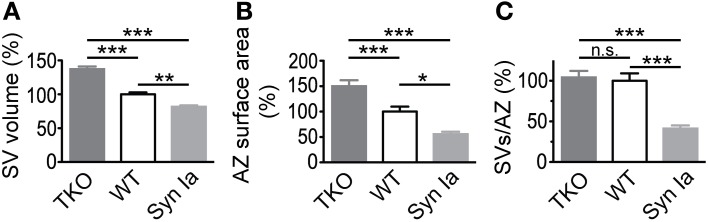
**SV diameter (A) and AZ surface area (B) scale with the relative abundance of synapsin isoforms in the calyx of Held.** Decreased absolute number of SV per AZ **(C)** in case of synapsin overexpression but not in synapsin triple knock-out compared to control/wild-type terminals. Parameters are normalized to control/wild-type values and One-Way ANOVA with Tukey-Kramer *post-hoc* test was used to determine statistical significance. Synapsin TKO data are taken from (Vasileva et al., 2012). ^*^P < 0.05; ^**^P < 0.01; ^***^P < 0.001.

We next examined the distribution of SVs proximal to the AZ, as these likely represent release-competent vesicles in the RRP. We estimated the surface area of the AZs by multiplying the total length by the thickness of the slice (Taschenberger et al., 2002). AZs from synapsin Ia-overexpressing calyces had smaller surface area compared to controls: 0.069 ± 0.006 μm^2^ vs. 0.124 ± 0.012 μm^2^ (Mann Whitney test, *P* < 0.0001; Figure 9H). There was a positive correlation between the AZ size and the number of associated SVs in EGFP-positive terminals (*r*^2^ = 0.81, *P* < 0.0001), while AZ size did not influence the number of SVs for synapsin Ia overexpression (*r*^2^ = 0.36, *P* = 0.0003; Figure 9I). The average SV density proximal to the AZ was decreased by synapsin Ia: 884.4 ± 60.7 SVs/μm^2^ AZ in EGFP and 596.3 ± 45.1 SVs/μm^2^ AZ in synapsin Ia-overexpressing calyces (Mann Whitney test *P* = 0.0009; Figure 10J). The distance between two neighboring SVs at the AZ was also shifted from 53.16 ± 0.87 nm in EGFP labeled synapses to 59.98 ± 1.56 nm in synapsin Ia overexpressing terminals (Mann Whitney test, *P* = 0.0002; Figure 9K). When SVs within the first 100 nm from the AZ surface area were examined, we found SVs distributed at variable distances from the AZ, albeit less in number within the clusters of synapsin Ia-overexpressing calyces, reflecting a decreased SV density (two-way ANOVA, *P* < 0.0001; Figures 9L,M). At distances from the AZ up to 400 nm synapsin Ia-overexpressing calyces still contained significantly less vesicles (Figure 9M).

In sum, ultrastructural data show that overexpression of synapsin Ia in the calyx of Held led to structural reorganization of SV clusters associated with AZs. Although the size of AZs and the number of proximal SVs decreased, the total number of SVs within the bulk volume of the presynaptic terminal remained the same as in control terminals. The remaining SVs seem to be sufficient to maintain basal synaptic transmission at normal levels and fail only when synapses are challenged with high-frequency repetitive stimulation.

## Discussion

We examined the localization pattern of synapsin isoforms in the calyx of Held presynaptic terminal, and correlated synaptic structure and function subsequent to perturbations of synapsin isoform expression *in vivo*, by combining quantitative fluorescence and EM with whole-cell patch-clamp recordings at the calyx of Held synapse. We found that overexpression of synapsin Ia increased short-term depression, accelerated recovery from depression, redistributed SV at AZ, and decreased both SV volume and AZ size.

### Synapsin isoform distribution in the calyx of held

3D immunohistochemistry revealed the distribution pattern of endogenous synapsin isoforms in the rat calyx of Held, prelabeled with membrane-anchored GFP (Dondzillo et al., 2010). All three synapsin genes were expressed in the calyx at postnatal day 16, with synapsins Ia, Ib, IIb and IIIa showing the most robust expression (Sudhof et al., 1989). Unexpectedly, synapsin IIa was not expressed in the calyx of Held. This isoform is prominent in many central glutamatergic synapses, and may peak later in adulthood, as reported for the cerebral cortex (Bogen et al., 2009). Contrary to its role in hippocampal cultures, where it maintains sustained transmission during high-frequency stimulation (Gitler et al., 2008), synapsin IIa is not required to support high-frequency transmission at the calyx at the maturational stage investigated here. Possibly other synapsin isoforms such as Ia or IIIa take over this function, although in cultured hippocampal neurons of synapsin triple-KO mice, these isoforms did not rescue synaptic depression (Gitler et al., 2008). This may be related to synapse-specific factors or due to differences in the abundance of synapsins achieved by the method of overexpression (transfection versus viral expression). Interestingly, synapsin IIa is lacking at other synapses with a high duty cycle such as Purkinje cell terminals in the deep cerebellar nuclei (Sudhof et al., 1989). In conclusion, it is intriguing to conjecture that the absence of synapsin IIa distinguishes synapses mediating high frequency transmission.

Within the calyx of Held the distribution pattern of the endogenous synapsin isoforms reaches beyond the boundaries of the vesicle clusters (Figure 3 here, and Figure 7 in Kempf et al., 2013), consistent with observations that synapsins exist in a state unbound to SVs (Cesca et al., 2010; Shupliakov et al., 2011). Using super-resolution STED microscopy, we recently showed that not all detected synapsin molecules overlapped with VGluT1 immunofluorescence (Kempf et al., 2013), consistent with the existence of synapsin-dependent and synapsin-independent components of the reserve pool (Vasileva et al., 2012).

Furthermore, synapsin Ia and synapsin Ib might be expressed in different subdomains (Figure 2). For example, different SV pools might be characterized by the presence of either synapsin Ia or synapsin Ib. Our results indicate that synapsin IIIa is a calyx-specific isoform within the MNTB, because it was not detected outside the boundaries of the presynaptic terminal (Figures 3I–K).

### Probing synapsin function by overexpression of GFP-synapsin I fusion proteins

Inherent to the approach of protein overexpression is the assumption that the increased abundance of the protein will affect function by saturating the reactions the protein is involved in. For proteins undergoing homo- and heterodimerization, like the synapsins (Hosaka et al., 1999), composition of dimers is expected to be dominated by the overexpressed protein. On the functional level, positive as well as negative changes can arise from protein overexpression. Ideally, the exact level of protein expression would be advantageous to know, however, in our study it was not possible to determine the exact amount because of technical limitations associated with our approach of studying a small number of calyx synapses perturbed *in vivo*.

For the interpretation of GFP-synapsin I overexpression experiments, it is important to know whether synapsin, when N-terminally fused to GFP or its variants, is functional. Although in principle it is difficult to be sure that fusion to GFP does not alter synapsin function, several lines of evidence strongly suggest that the known properties of synapsin I isoforms are retained in the GFP fusion proteins: (1) GFP-synapsin is properly targeted to the presynaptic terminals as shown in Figure 4 and by Gitler et al. (2004a); (2) EGFP-synapsin Ia was used to examine real-time dynamics of synapsin in response to AP firing in rat hippocampal neuronal cultures (Chi et al., 2001); (3) Synapsin Ia, N-terminally fused to ECFP, was used to demonstrate that cAMP-modulated phosphorylation of synapsin Ia controls the distribution of SVs in the growth cones of rat hippocampal neurons (Bonanomi et al., 2005); (4) EGFP-synapsin Ia/mCherry-synapsin Ia was used to examine the effects of synapsin I phosphorylation on post-tetanic potentiation in mouse glutamatergic synapses in the synapsin I knock-out background. Expression of the fusion proteins fully rescued PTP (Valente et al., 2012); (5) Finally, full rescue was found in cultured hippocampal neurons of synapsin TKO mice after viral overexpression of GFP-synapsin IIa (Shulman and Gitler, unpublished results) as opposed to a partial rescue achieved with overexpression by transfection (Gitler et al., 2008).

### Redistribution of SVs by overexpression of synapsin I isoforms

Our findings strongly suggest that synapsin I isoforms mediate SV distribution within the presynaptic terminal. Both on the scale of entire presynaptic terminals, and on the scale of clusters associated with single AZs, overexpression of synapsin Ia causes vesicle redistribution (Figures 5, 9). Overexpression of either synapsin Ia or synapsin Ib might lead to less efficient immobilization of SVs, leading to diffusive vesicle relocation within the available volume of the calyx of Held. The excess of synapsin molecules may increase the chance that synapsins form dimers without being linked to SVs (Gitler et al., 2004a), thereby interfering with SV clustering at the AZ. Consistent with our results, overexpression of synapsin in *Aplysia* sensorimotor neuron co-cultures has been reported to enlarge the reserve pool of SVs (Fioravante et al., 2007).

Overexpression of synapsin Ia did not cause noticeable alterations in the overall ultrastructure of the calyceal segments examined with EM (Figure 8). However, SV clusters closely associated with AZs contained fewer SVs. The low number of docked vesicles found here (Figure 9) coincides with the maturational decrease in docked vesicle number (Taschenberger et al., 2002). While synapsins are required to maintain a large distal SV pool without affecting the AZ-proximal pool (Vasileva et al., 2012), when overexpressed they may interfere with an optimal cross-linking of SV through dimer formation (Hosaka and Sudhof, 1999; Gitler et al., 2004a). The more pronounced reduction in AZ-proximal pools we observed might suggest that excess of synapsin interferes with another molecular entity recruiting SVs to the AZ cluster. At small central nervous system synapses recycling vesicles are preferentially arranged near the AZ, and this segregation is abolished by actin stabilization (Marra et al., 2012). Synapsin I is able to nucleate, bind to, and bundle actin (Petrucci and Morrow, 1987); hence, overexpressed synapsin I may interfere with the dynamic formation of the actin matrix (Bloom et al., 2003). Additionally, the reduced size of SVs may favor their loss near the AZ because of an increased mobility, or reduced capture by the local cytoskeleton. Alternatively, the reduction of the AZ surface area may result in a smaller associated SV cluster assuming a homeostatic mechanism that matches AZ size and size of the SV cluster (Schikorski and Stevens, 1997). However, the robust SV/AZ surface-size correlation found for control AZs was lost upon synapsin Ia overexpression (Figure 9I), arguing for a stronger synapsin-specific effect on the integrity of the vesicle cluster than expected merely from a homeostatic modulatory mechanism.

### Synapsin Ia overexpression alters the size of synaptic vesicles and active zones

Overexpression of synapsin Ia led to alterations in the size of SVs and AZ—SVs were smaller and AZ surface area was halved (Figure 9). Interestingly, the opposite phenotype was observed after deletion of all synapsin isoforms—larger SVs and AZs (Vasileva et al., 2012). This strongly indicates that the abundance of synapsin proteins regulates both SV and AZ size (Figure 10). A decreased SV diameter with increased synapsin concentration might be related to the stabilizing effect of synapsins on vesicle lipid bilayers (Pera et al., 2004). Another possibility is that vesicle reuptake and recycling upon exocytosis or SV biosynthesis are impaired leading to smaller SVs. Notably, the synapsin-mediated changes in SV diameter did not translate into altered spEPSC amplitudes (see Discussion in (Vasileva et al., 2012). Not much is known about the regulation of AZ size. In the absence of synapsins, the surface area of small hippocampal boutons was altered, yet the size of cerebellar granule cell terminals remained unchanged (Takei et al., 1995; Siksou et al., 2007). To the contrary, Kielland et al. reported an increased terminal area in corticothalamic synapses in synapsin I and II knock outs (Kielland et al., 2006). Interestingly, a recent study showed a direct correlation of the active zone size with the number of active zone proteins and docked SVs (Holderith et al., 2012). Similarly, the size of the AZ and the number of associated SVs may be maintained at a constant ratio (Schikorski and Stevens, 1997). These findings argue for the existence of mechanisms that tightly regulate the composition of AZs, while a mechanism for synapsin-mediated regulation of AZ size remains elusive. A functional link of synapsins to the AZ protein piccolo has been suggested (Leal-Ortiz et al., 2008) and therefore may represent a first lead to address this issue.

### Synapsin overexpression increases RRP depletion and refilling kinetics

Overexpression of synapsin I isoforms caused a significant acceleration of the decay times and produced deeper steady-state depression, consistent with previous work (Gitler et al., 2004b; Sun et al., 2006; Gabriel et al., 2011; Vasileva et al., 2012). It also resulted in smaller SV clusters near the AZs, leading to accelerated and enhanced short-term depression. This is consistent with reduced vesicle number per AZ and fewer SVs available to refill the RRP during repetitive synaptic activity. Both synapsin I isoforms thus appear to participate in organizing the vesicle pool of the calyx, controlling the activity-dependent transition of SVs from the reserve pool into the recycling pool or RRP.

Recovery from short-term depression was accelerated in calyces overexpressing synapsin Ia, but not synapsin Ib. This appears inconsistent with the accelerated short-term depression observed most strongly after synapsin Ia overexpression (Figure 6), yet the stronger extent of vesicle pool depletion observed upon synapsin Ia overexpression may accelerate the fast component of recovery. Faster RRP recovery might be explained by compromised tethering of SVs to the cytoskeleton in the synapsin Ia-overexpressing terminals. Presynaptic studies at immature rats have shown that recovery from synaptic depression is frequency-dependent. The recovery after moderate stimulation (10–100 Hz) normally follows a monoexponential time course, but overexpression of either synapsin I isoforms introduced an additional fast component, potentially linked to the observed increase in release probability (Figure 6L). The fast component of recovery reflects a calcium-dependent increase in SV replenishment (Wang and Kaczmarek, 1998) through a possible Ca^2+^—calmodulin—Munc13-1 mechanism (Lipstein et al., 2013). Additionally, (Lee et al., 2012) showed that the recruitment of SVs into the fast-releasing vesicle pool strongly depends on polymerization of actin. Therefore, an increase in the release probability of “reluctant” rapidly replenishing SVs (Wu and Borst, 1999; Sakaba and Neher, 2001b; Hosoi et al., 2007; Muller et al., 2010) through synapsin-actin interactions (Shupliakov et al., 2011) might be involved in the speeding of the recovery process. The accelerated recovery from depression due to synapsin Ia overexpression suggests that this isoform fulfills an additional function within the recycling pool, possibly related to Ca^2+^-modulated SV endocytosis or recycling (Bloom et al., 2003; Coleman et al., 2008). In fact, a direct connection between endocytosis and synapsin dispersion within the presynaptic terminal has been shown previously (Orenbuch et al., 2012). Hence, synapsin Ia may potentiate an existing mechanism that translates the Ca^2+^ signal into faster SV retrieval and recycling.

## Author contributions

Study concept and design: Mariya Vasileva, Robert Renden, Thomas Kuner, Daniel Gitler. Acquisition of data: Mariya Vasileva, Robert Renden, Heinz Horstmann. Analysis and interpretation of data: Mariya Vasileva, Robert Renden, Thomas Kuner. Drafting of the manuscript: Mariya Vasileva, Robert Renden, Thomas Kuner. Critical revision of the manuscript for important intellectual content: Thomas Kuner, Daniel Gitler. Obtained funding: Thomas Kuner, Daniel Gitler, Robert Renden. Study supervision: Thomas Kuner.

### Conflict of interest statement

The authors declare that the research was conducted in the absence of any commercial or financial relationships that could be construed as a potential conflict of interest.
